# Molecular and biochemical characterization of key enzymes in the cysteine and serine metabolic pathways of *Acanthamoeba castellanii*

**DOI:** 10.1186/s13071-018-3188-7

**Published:** 2018-11-26

**Authors:** Duo Wu, Meng Feng, Zhi-xin Wang, Ke Qiao, Hiroshi Tachibana, Xun-jia Cheng

**Affiliations:** 1 Department of Medical Microbiology and Parasitology, School of Basic Medical Sciences, Fudan University, Shanghai, 200032 China; 2 Department of Infectious Diseases, Tokai University School of Medicine, Isehara, Kanagawa 259-1193 Japan

**Keywords:** Cysteine synthase, Cysteine biosynthesis, Glycerate dehydrogenase, Glycolysis pathway, Drug target

## Abstract

**Background:**

*Acanthamoeba* spp. can cause serious human infections, including *Acanthamoeba* keratitis, granulomatous amoebic encephalitis and cutaneous acanthamoebiasis. Cysteine biosynthesis and the L-serine metabolic pathway play important roles in the energy metabolism of *Acanthamoeba* spp. However, no study has confirmed the functions of cysteine synthase (*Ac*CS) in the cysteine pathway and phosphoglycerate dehydrogenase (*Ac*GDH) or phosphoserine aminotransferase (*Ac*SPAT) in the non-phosphorylation serine metabolic pathway of *Acanthamoeba*.

**Methods:**

The *AcCS*, *AcGDH* and *AcSPAT* genes were amplified by PCR, and their recombinant proteins were expressed in *Escherichia coli*. Polyclonal antibodies against the recombinant proteins were prepared in mice and used to determine the subcellular localisation of each native protein by confocal laser scanning microscopy. The enzymatic activity of each recombinant protein was also analysed. Furthermore, each gene expression level was analysed by quantitative PCR after treatment with different concentrations of cysteine or L-serine.

**Results:**

The *AcCS* gene encodes a 382-amino acid protein with a predicted molecular mass of 43.1 kDa and an isoelectric point (pI) of 8.11. The *AcGDH* gene encodes a 350-amino acid protein with a predicted molecular mass of 39.1 kDa and a pI of 5.51. The *AcSPAT* gene encodes a 354-amino acid protein with a predicted molecular mass of 38.3 kDa and a pI of 6.26. Recombinant *Ac*CS exhibited a high cysteine synthesis activity using O-acetylserine and Na_2_S as substrates. Both GDH and SPAT catalysed degradation, rather than synthesis, of serine. Exogenous L-serine or cysteine inhibited the expression of all three enzymes in a time- and dose-dependent manner.

**Conclusions:**

This study demonstrated that *Ac*CS participates in cysteine biosynthesis and serine degradation *via* the non-phosphorylation serine metabolic pathway, providing a molecular basis for the discovery of novel anti-*Acanthamoeba* drugs.

**Electronic supplementary material:**

The online version of this article (10.1186/s13071-018-3188-7) contains supplementary material, which is available to authorized users.

## Background

Free-living amoebae of the genus *Acanthamoeba* are widespread protozoans that exist as vegetative trophozoites and dormant cysts during their life-cycle [1]. Some *Acanthamoeba* species cause accidental infections, such as *Acanthamoeba* keratitis [2], fatal granulomatous amoebic encephalitis or cutaneous infections in immunosuppressed individuals [3]. The number of reported cases worldwide is increasing every year. Despite the medical importance of these parasites, however, few antimicrobial agents are available for treatment [4].

Amino acid metabolic pathways are potential drug targets in some infectious diseases. L-cysteine is a sulphur-containing amino acid that plays an important role in the structure, stability, catalytic activity and regulation of numerous proteins [5–9]. This amino acid is also involved in the synthesis of many other important biomolecules, such as glutathione, thiamine, taurine, lipoic acid, biotin and coenzyme A. Therefore, its synthetic pathways represent an attractive drug target for treatment. Cysteine can be generated from methionine through the transsulphuration pathway in mammals [5] and fungi [10] or from serine and inorganic sulphide. The latter pathway is also known as sulphur assimilation and occurs in bacteria [11], plants [12] and a few protozoa, such as *Entamoeba histolytica* [13] and *Trypanosoma cruzi* [14]. This pathway comprises two catalytic steps that are initiated by serine acetyltransferase (SAT) to form O-acetylserine (OAS) from L-serine and acetyl-coenzyme A. OAS reacts with sulphide to generate cysteine, and this step is catalysed by cysteine synthase (CS, OAS thiolyase) [15].

To date, the presence of the sulphur assimilation/cysteine biosynthetic pathway in *A. castellanii* remains unclear. However, in a previous study, we confirmed the existence of a phosphorylated serine biosynthetic pathway in this organism [16]. Phosphoglycerate dehydrogenase (PGDH) catalyses the first step of this pathway by oxidising 3-phosphoglycerate to 3-phosphohydroxypyruvate (3-PHP) using NAD^+^/NADH as a cofactor. Phosphoserine aminotransferase (PSAT) catalyses the reversible conversion of 3-PHP to phosphoserine, which is then dephosphorylated by phosphoserine phosphatase to form L-serine [17]. Serine is a cysteine precursor in the sulphur assimilation/cysteine biosynthetic pathway of nearly all organisms, including protozoa. Hence, serine metabolic pathways are important in the regulation of cysteine levels.

To understand the role of serine metabolism and sulphur-containing cysteine biosynthesis in *A. castellanii*, we searched the Kyoto Encyclopaedia of Genes and Genomes database and created a list of potential enzymes based on the available pathway information. Then, we successfully cloned the gene of D-glycerate dehydrogenase (GDH) and serine-pyruvate aminotransferase (SPAT) from *A. castellanii* and demonstrated that this amoeba possesses phosphorylated and nonphosphorylated pathways for serine metabolism. We extended this work by cloning the *CS* gene of *A. castellanii*. However, we could not identify the gene for SAT, which is absent in this organism because no homologous genes could be found for it. This finding demonstrates that *A. castellanii* possesses a cysteine biosynthetic pathway involving CS but not SAT. Humans lack CS; thus, this parasitic enzyme could be a target for the rational design and screening of anti-*Acanthamoeba* agents. To the best of our knowledge, this work is the first to report on the non-phosphorylated pathway for serine metabolism and CS-mediated cysteine biosynthesis in a free-living amoeba.

## Methods

### Chemicals

All chemicals were of analytical grade and purchased from Sigma-Aldrich (Shanghai, China), unless stated otherwise.

### Amoeba culture

*Acanthamoeba castellanii* (strain ATCC30011) was obtained from the American Type Culture Collection, and trophozoites were routinely grown axenically in peptone-yeast-glucose (PYG) medium [18]. Cultures were incubated at 26 °C, and trophozoites were harvested in the late log phase after subculture for 72 h.

### Cloning and expression of genes

Total *A. castellanii* RNA was extracted from trophozoites using RNeasy® Plus Mini Kit (Qiagen, Hilden, Germany), and complementary DNA (cDNA) was synthesised using a PrimeScript® 1st strand cDNA synthesis kit (Takara, Kusatsu, Japan). The *CS*, *GDH* and *SPAT* genes were amplified by PCR using the primers listed in Table 1. PCR was performed in a 9902 Veriti 96-well Thermal Cycler (Applied Biosystems, Waltham, USA) (94 °C for 3 min; 35 cycles of 94 °C for 15 s, 55 °C for 30 s and 72 °C for 1 min; followed by 72 °C for 5 min). The amplified PCR product was purified and ligated into a pMD19-T Vector (Takara), and the nucleotide sequence was obtained by automated sequencing. The correct plasmids were amplified with primers containing *Nde*I and *Bam*HI restriction site sequence, and the PCR products were ligated into pET19b. The sequences of all constructs were confirmed on both strands and analysed with Vector NTI software (Invitrogen, Waltham, USA). Plasmids were transformed into *E. coli* BL21(DE3) pLysS for protein expression, and recombinant CS, GDH and SPAT proteins were purified using a QIA Express kit in accordance with the manufacturer’s instructions. The purity and mass of each protein was determined by sodium dodecyl sulphate polyacrylamide gel electrophoresis. The concentrations of recombinant proteins were measured using a protein assay (Bio-Rad, Hercules, USA).

**Table 1:** Primers used for amplification of *Acanthamoeba castellanii* genes

Gene	Primer	Sequence (5'-3')
CS	CS-ORF-S	ATGAAGCACATGACCCCACGC
	CS-ORF-AS	TTATTGGAGGTACTTGTCGGGAAGG
	CS-qPCR-S	CTCCGTCCTCTATTCGTAC
	CS-qPCR-AS	ATGCTCATTGTTTCTTCGT
GDH	GDH-ORF-S	ATGGACAGCGGTGCGAG
	GDH-ORF-AS	TCAAGGATTGACGCGCATCA
	GDH-qPCR-S	GGTCGCATTGGCAAGAGG
	GDH-qPCR-AS	GCGTCGAGTCCGTGAGGTT
SPAT	SPAT-ORF-S	ATGGAGGCTGACCGTCCGCTGCT
	SPAT-ORF-AS	TTAGAGCTTGTTGGGCAGTGCTG
	SPAT-qPCR-S	TCGCCCGACTTTATCAACCTCT
	SPAT-qPCR-AS	TGCGCACCGAACGACTCCAA

### Preparation of polyclonal antibodies and dot blot analysis

Each purified recombinant protein was injected intraperitoneally into BALB/c mice. In brief, 50 μg of protein was mixed with Freund’s complete adjuvant for the first injection, and Freund’s incomplete adjuvant was used for the next two immunisations at 2-week intervals. One week after the final injection, serum was collected from the mice. Total crude protein from *A. castellanii* trophozoites (5 μg) and aliquots of purified CS, GDH and SPAT (1 μg) were blotted on a nitrocellulose membrane [19]. Recombinant *Babesia microti* merozoites (BMSA) [20] and antigen extracts from *Dermatophagoides farinae* (Derf 2; stocks from our laboratory, 1 μg) were used as negative controls. Filter strips were blocked with 3% skimmed milk powder in PBS and then incubated with 1:50 diluted anti-*Ac*CS, anti-*Ac*GDH or anti-*Ac*SPAT mouse sera. Horseradish peroxidase-conjugated (HRP) goat anti-mouse IgG (Cappel, Chester, USA) was used as a secondary antibody. Proteins were visualised using an enhanced HRP-DAB substrate detection kit (Tiangen Biotech, Beijing, China).

### Confocal microscopy

*Acanthamoeba castellanii* trophozoites at a density of 2 × 10^5^ cells/ml were used for confocal microscopy. To assess colocalisation of native *Ac*GDH and *Ac*SPAT, the trophozoites were incubated for 1 h with a mouse anti-*Ac*GDH polyclonal antibody (1:50 dilution with 3% skimmed milk powder in PBS), followed by 1 h with an Alexa Fluor 488 goat anti-mouse IgG_1_ (H + L; Abcam, Cambridge, USA) as a secondary antibody. Next, an anti-*Ac*SPAT mouse polyclonal antibody (1:50 dilution) was used to react with native *Ac*SPAT for 1 h, followed by an Alexa Fluor 568 goat anti-mouse IgG_2b_ (H + L; Abcam) as a secondary antibody for 1 h. The cells were also stained with 0.25-mg/ml 4',6-diamidino-2-phenylindole (DAPI) and 1.25-mg/ml 1,4-diazabicyclo[2.2.2]octane (DABCO, in 10 % glycerol-PBS) for 10 min. For *Ac*CS localisation, the cells were incubated for 1 h with anti-*Ac*CS mouse polyclonal antibody (1:50 dilution) used as a primary antibody, followed by 1 h with an Alexa Fluor 488 goat anti-mouse IgG_1_ (H + L; Abcam) used as a secondary antibody. Propidium iodide was used to stain nuclei. Cell suspensions containing 2 × 10^4^ cells were placed onto glass slides, mounted with coverslips and examined using a Leica TCS SP8 (Wetzlar, Germany) microscope.

### Cysteine synthase assay

CS activity was measured as described by Westrop et al. [21]. Briefly, reaction mixtures contained 50 mM Tris-HCl (pH 7.5), 0.2 mM pyridoxal 5'-phosphate, 20 mM OAS, 2 mM Na_2_S and the corresponding recombinant CS protein. Reactions were stopped by adding 50 μl of glacial acetic acid (> 99.9%), and the formation of cysteine was quantified by the method described by Gaitonde [22]. The K_m_ for OAS was determined using 3 mM Na_2_S and by varying the concentration of OAS between 10 and 100 mM. The K_m_ of Na_2_S was determined using 30 mM OAS and by varying the concentrations of Na_2_S between 0.1 and 12.8 mM.

### Glycerate dehydrogenase assay

GDH activity was determined in both forward and reverse directions [13]. Briefly, the reactions contained 20 mM sodium phosphate buffer (pH 6.5), 300 mM NaCl, 0.2 mM NADPH, 0.2 mM dithiothreitol (DTT), 10 mM lithium β-hydroxypyruvic acid (hydroxypyruvate, HP) and the purified protein. The K_m_ for HP was determined using 1.4 mM NADPH and by varying the concentration of HP between 7.5 and 90 mM. The K_m_ of NADPH was determined using 22.5 mM HP and by varying the concentrations of NADPH between 0.4 and 8 mM. In the reverse reactions, the reactions contained 50 mM Tris-HCl buffer (pH 8.5), 300 mM NaCl, 0.2 mM DTT, 10 mM NADP^+^, 10 mM hemicalcium D-glyceric acid (glycerate) and the protein. The K_m_ for glycerate was determined using 30 mM NADP^+^ and by varying the concentration of glycerate between 100 and 1000 mM. The K_m_ of NADP^+^ was determined using 150 mM glycerate and by varying the concentrations of NADP^+^ between 20 and 240 mM.

### Serine-pyruvate aminotransferase assay

Serine-pyruvate aminotransferase activity was detected as described by Snell et al. [23]. Briefly, reaction mixtures contained 50 mM Tris-HCl buffer (pH 8.5), 20 mM L-serine, 20 mM sodium pyruvate and 40 μM pyridoxal 5′-phosphate (PLP), and the reaction was initiated by adding purified SPAT protein. After incubation for 30 min, 70% (w/v) HClO_4_ was added to stop the reactions. Subsequently, active *Ac*GDH proteins were used to assess HP generation. The K_m_ for serine was determined using 4 mM pyruvate and by varying the concentration of serine between 0.125 and 5 mM. The K_m_ of pyruvate was determined using 0.5 mM serine and by varying the concentration of pyruvate between 2 and 25 mM.

The kinetic constants reported for all assays are the means of at least three independent determinations, and K_m_ and V_max_ values were estimated using Michaelis-Menten and Lineweaver-Burk plots in conjunction with non-linear regression using GraphPad Prism v.5.01. Turnover numbers (k_cat_ s^-1^) were calculated using the molecular mass of one subunit of each protein.

### Treatment with L-serine or cysteine and quantitative PCR analysis

Trophozoites were seeded in a 24-well plate (Eppendorf, Hamburg, Germany) at a density of 1 × 10^5^ cells/ml in PYG medium [24]. L-serine or cysteine were added at concentrations of 10, 20 and 40 mM, followed by incubation at 26 °C. Trophozoites were collected after 12 and 24 h of incubation, and total RNA was extracted. Real-time quantitative PCR was performed to analyse *Ac*CS, *Ac*GDH and *Ac*SPAT gene expression. The primers used are shown in Table 1.

### Statistical analysis

All statistical analysis was performed using GraphPad 5 software (San Diego, CA, USA). Significance was calculated by one-way ANOVA analysis followed by a Tukey test. Data were expressed as the mean ± standard deviation (SD) and at least three independent experiments were performed for each sample. *P* < 0.05 was considered to be significant.

## Results

### Cloning of the genes encoding *A. castellanii* CS, GDH and SPAT

The sequences of GDH and SPAT with the accession numbers LC154947 and LC154948, respectively, have been submitted to the GenBank database, whereas the sequence of CS is still under submission. The *CS* gene contains a 1149-bp open reading frame (ORF) that encodes a 382-amino acid protein with a predicted molecular mass of 43.1 kDa and an isoelectric point (pI) of 8.11. The GDH gene contains a 1053-bp ORF that encodes a 350-amino acid protein with a predicted molecular mass of 39.1 kDa and a pI of 5.51. The SPAT gene contains a 1062-bp ORF encoding a 354-amino acid protein with a predicted molecular mass of 38.3 kDa and a pI of 6.26.

### Reactivity of mouse polyclonal antibodies to *Ac*CS, *Ac*GDH and *Ac*SPAT

**Fig. 1:**
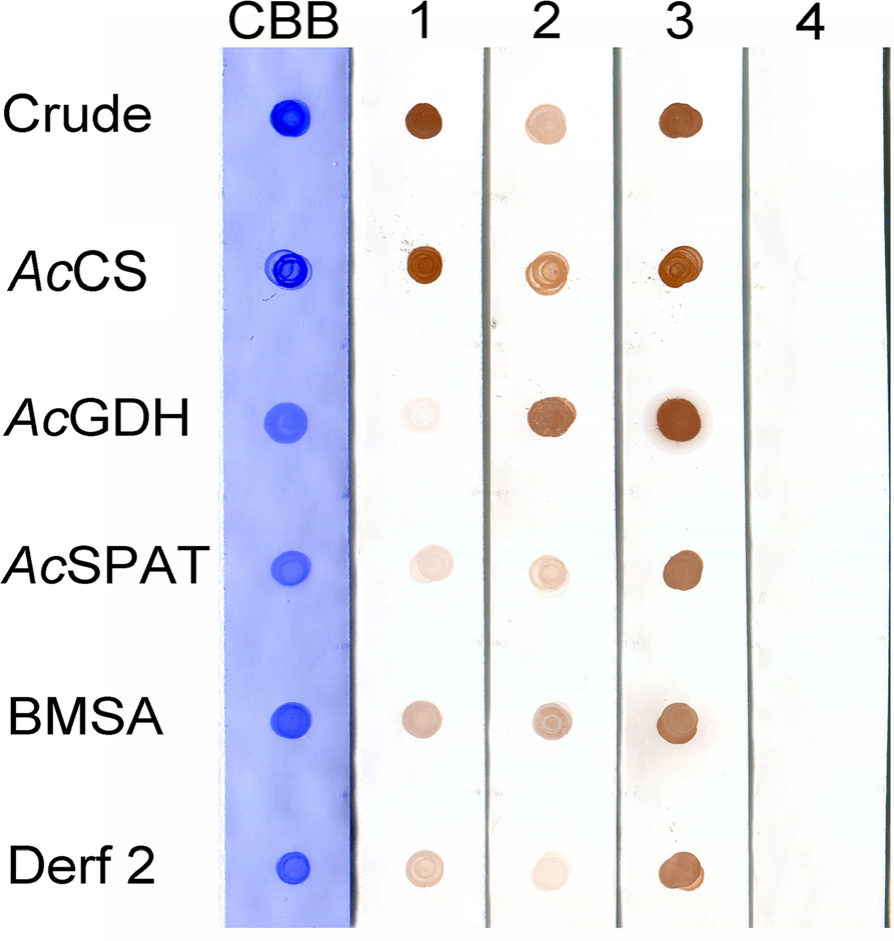
Dot blot analysis of reactivity of mouse polyclonal antibodies to *Ac*CS, *Ac*GDH or *Ac*SPAT. Strips were spotted with extracted crude proteins from trophozoites (5 μg) and with each purified recombinant protein (1 μg). BMSA and Derf 2 were used as negative controls. One strip was stained with Coomassie brilliant blue. Strips 1, 2 and 3 were treated with anti-*Ac*CS, anti-*Ac*GDH and anti-*Ac*SPAT mouse polyclonal serum, respectively. Strip 4 was treated with normal mouse serum as a negative control

The reactivities of polyclonal antibodies raised against *Ac*CS, *Ac*GDH and *Ac*SPAT were assessed by a dot blot analysis (Fig. 1). Crude (5 μg) and recombinant proteins (1 μg) were spotted on a nitrocellulose membrane, and the crude antigens in strips 1, 2 and 3 exhibited strong immunoreactivity, indicating that the polyclonal antibodies recognised the respective proteins in trophozoites. However, the anti-*Ac*SPAT polyclonal antibody exhibited cross-reactivity with both CS and GDH recombinant proteins. No immunoreactivity was observed in normal mouse serum to any of the spotted antigen.

### Localisation of native CS, GDH and SPAT in *A. castellanii*

**Fig. 2:**
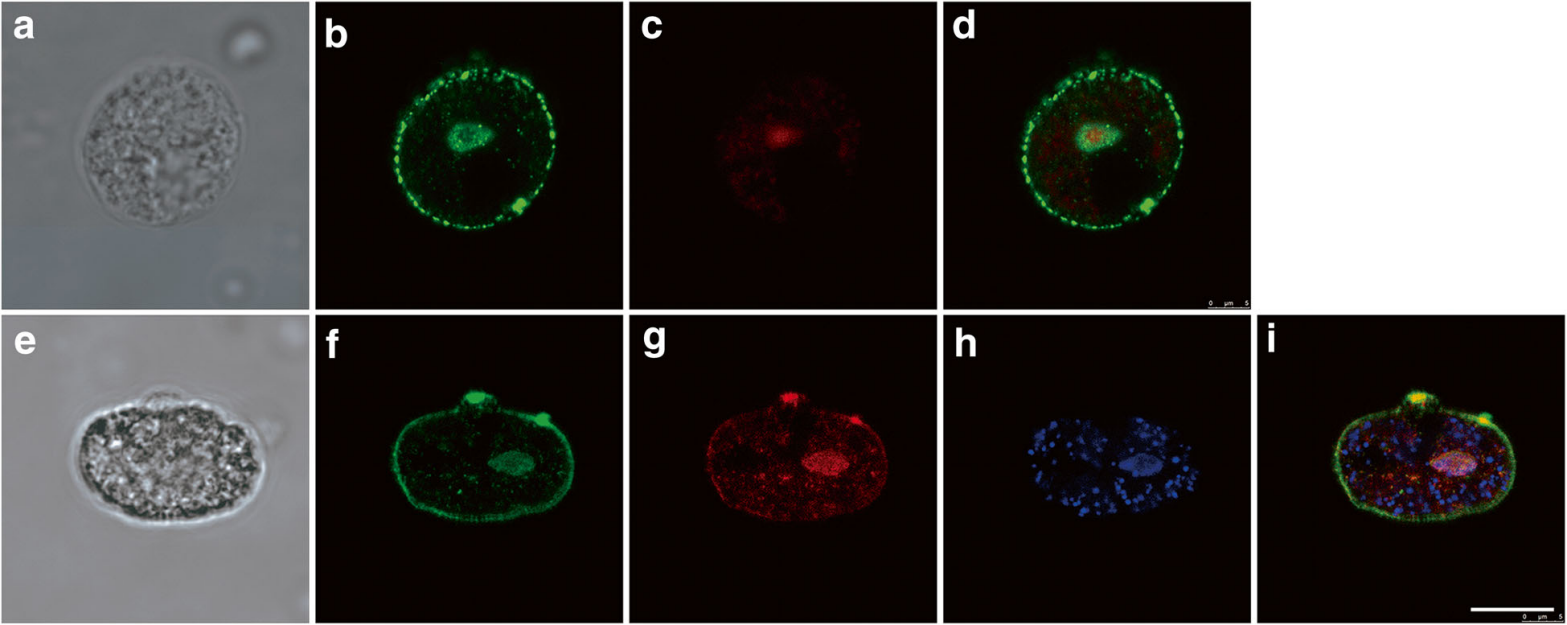
Localization of native CS, DGH and SPAT in *Acanthamoeba* trophozoites by confocal microscopy. **a-d** Localisation of *Ac*CS, **e-i** colocalisation of *Ac*GDH and *Ac*SPAT in trophozoites. **a**, **e** Differential interference contrast microscopy. **b** Staining with a polyclonal antibody against *Ac*CS. **c** Nuclear staining with propidium iodide. **d** Merged image of **b** and **c**. **f** Staining with a polyclonal antibody against *Ac*GDH. **g** Staining with a polyclonal antibody against *Ac*SPAT. **h** Nuclear staining with DAPI. **i** Merged image of **f**, **g** and **h**. *Scale-bar*: 10 μm

To determine the localisation of native CS, GDH and SPAT in *A. castellanii*, confocal laser scanning microscopy was performed using anti-CS, anti-GDH and anti-SPAT mouse polyclonal antibodies (Fig. 2). *Ac*CS was found to be localised to the inner part of the cell membrane (Fig. 2b), *Ac*GDH and *Ac*SPAT appeared to be colocalised (Fig. 2i) to the inner part of the cell membrane and to the membranes surrounding the nucleus.

### Kinetic properties of *Ac*CS, *Ac*GDH and *Ac*SPAT

**Fig. 3:**
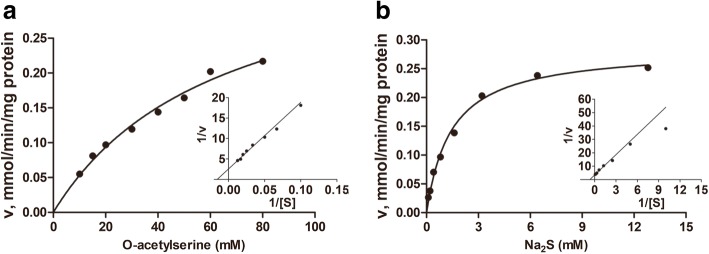
Michaelis-Menten and Lineweaver-Burk double-reciprocal plots for the substrates of CS of *A. castellanii*. The enzyme assay was performed by varying one substrate concentration in the presence of a saturating concentration of the other substrate (see the experimental section). Panels **a** and **b** show the Michaelis-Menten plots obtained for cysteine and Na_2_S as the variable substrates, respectively. The corresponding Lineweaver-Burk plots are depicted as insets on the right side of each panel

To assess the potential role of CS in cysteine biosynthesis, its sulphydrylase activity was tested. Recombinant *Ac*CS could readily catalyse cysteine synthesis using OAS and Na_2_S as substrates and showed a high affinity for both substrates (Fig. 3). The enzyme was highly active in the pH range of 7.0 to 7.5.

The *Ac*GDH activity was measured in both forward and reverse directions. In the forward reaction, GDH catalyses the reduction of HP to glycerate using NADPH as a cofactor, whereas in the reverse reaction, it converts glycerate to HP using NADP^+^ as a cofactor. Recombinant *Ac*GDH exhibited a higher affinity for HP than for glycerate as K_m_ for glycerate was 10-fold higher than K_m_ for HP (Table 2). Concordantly, the apparent K_m_ of NADP^+^ was higher than K_m_ of NADPH; however, the specific activity was 10-fold higher with NADP^+^ than with NADPH. In the forward reaction, *Ac*GDH exhibited a high activity in the pH range of 6.0–6.5, and in the reverse reaction, *Ac*GDH showed the highest activity in the pH range of 7.8–8.5. These data suggest that *Ac*GDH specifically catalyses the conversion HP to glycerate.

**Table 2:** Kinetic parameters of *Ac*CS, *Ac*GDH and *Ac*SPAT

Recombinant proteins	Substrate/cofactor	*Km* (mM)	Specific activity (mmol/min per mg of protein)	*kcat* (^-^s)
*Ac*CS	O-acetylserine	0.39 ± 0.02	63.92 ± 6.33	
	Na_2_S	1.44 ± 0.08	0.28 ± 0.01	2.36 × 10^4^ ± 978
*Ac*GDH	Hydroxypyruvate	17.93 ± 1.71	26.50 ± 0.85	
	NADPH	1.01 ± 0.06	2.05 ± 0.04	9.5 × 10^3^ ± 297
	Glycerate	213.80 ± 19.77	20.48 ± 0.65	
	NADP+	19.90 ± 0.74	20.85 ± 0.17	1.38 × 10^4^ ± 273
*Ac*SPAT	Pyruvate	5.26 ± 0.40	6.98 ± 0.18	
	Serine	0.72 ± 0.03	13.17 ± 0.19	6.66 × 10^3^ ± 122

The enzyme assays were performed by varying one substrate concentration in the presence of a saturating concentration of the other substrate (see the experimental section). The k_cat_ values reported are the means calculated from the V_max_ values obtained for both substrates for each enzyme (the k_cat_ for *Ac*CS was obtained using OAS and Na_2_S; the k_cat_ for the forward reaction of *Ac*GDH was obtained using hydroxypyruvate and NADPH, whereas that for the reverse reaction was obtained using glycerate and NADP^+^; the k_cat_ for *Ac*SPAT was obtained using pyruvate and serine). Reactions were performed at 37 °C and results represent means ± SD from triple experiments

SPAT catalyses the conversion of pyruvate to HP using L-serine as an amine donor; in the reverse reaction, it converts HP to pyruvate using alanine as an amine donor. However, we could not test for the conversion of HP to pyruvate because the addition of lactate dehydrogenase would be required to monitor HP generation. The optimal pH for *Ac*SPAT was 8.0–8.5, and its specific activity was 6.99 and 13.17 mmol/min/mg protein with pyruvate and serine, respectively, suggesting that *Ac*SPAT could transform both serine and pyruvate.

### Effects of cysteine and L-serine on the gene expression levels of key enzymes

**Fig. 4:**
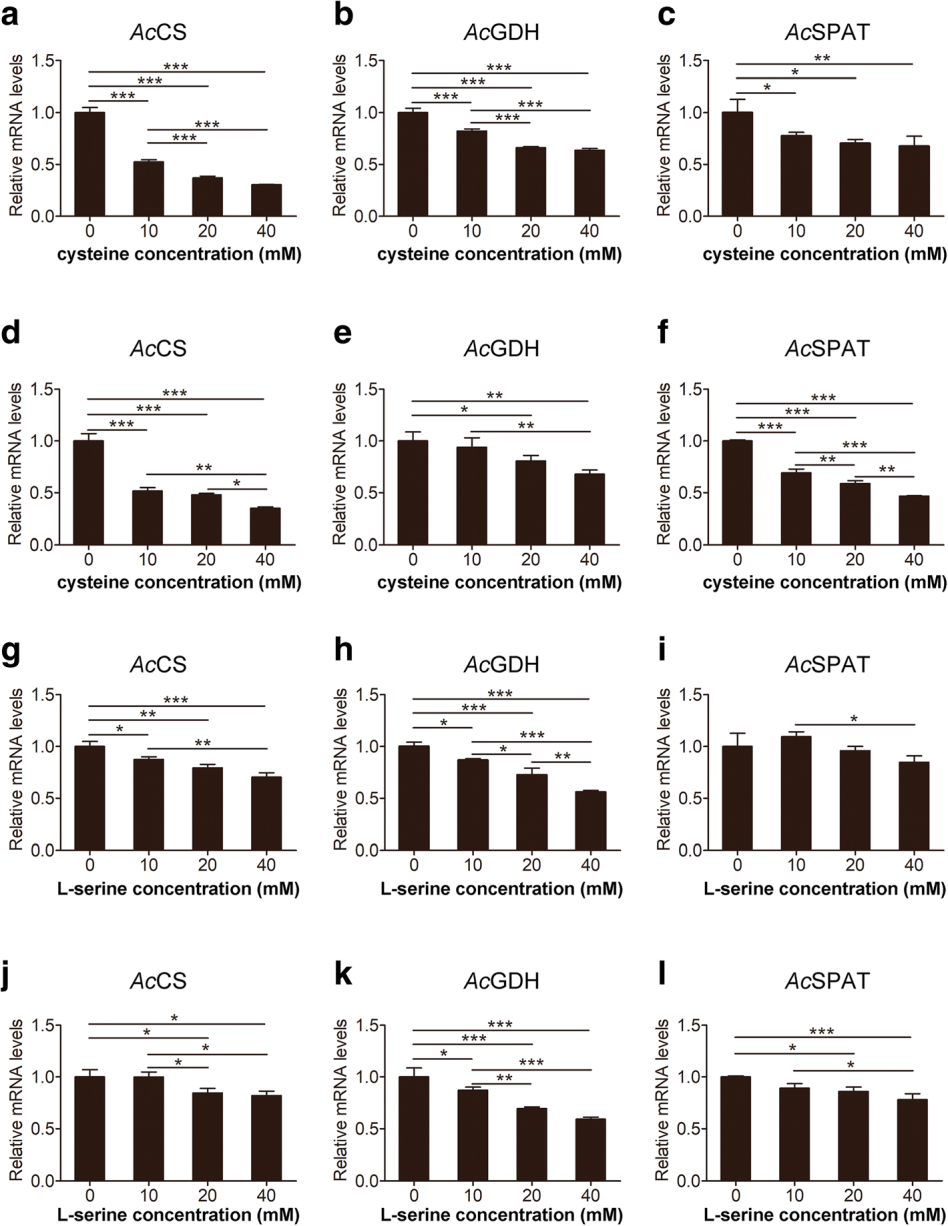
Effect of cysteine (**a-f**) or L-serine (**g-l**) on *CS*, *GDH* and *SPAT* gene expression in *A. castellanii* trophozoites. **a-c** Gene expression levels after treatment with different concentrations of cysteine (10, 20 and 40 mM) for 12 h. **d-f** Gene expression levels after treatment with cysteine for 24 h. **g-i** Gene expression levels after treatment with different concentrations of L-serine (10, 20 and 40 mM) for 12 h. **j-l** Gene expression levels after treatment with serine for 24 h. As a control, trophozoites were cultured in PYG medium with no amino acid treatment. Vertical bars indicate SD. *P < 0.05, **P < 0.01 and ***P < 0.001 by one-way ANOVA

To obtain further evidence that *Ac*CS is involved in cysteine biosynthesis and to verify that *Ac*GDH and *Ac*SPAT participate in the serine degradation pathway in *A. castellanii*, exogenous cysteine or serine were added to the culture medium of trophozoites and subsequent gene expression levels of these enzymes were assessed using quantitative PCR (Fig. 4). *Ac*CS, *Ac*GDH and *Ac*SPAT expression was significantly downregulated by cysteine in a dose-dependent manner after treatment for 12 or 24 h, and no significant difference in their expression was observed between these two time points. *Ac*CS and *Ac*GDH expression was also significantly inhibited by L-serine in a dose-dependent manner after incubation for 12 or 24 h. However, *Ac*SPAT expression slightly increased at treatment with 10 mM serine for 12 h but decreased at higher concentrations or after 24 h of treatment.

## Discussion

This study is the first to report on the cysteine synthetic and non-phosphorylated serine metabolic pathways in *A. castellanii*. The properties of *Ac*CS presented here provide molecular insights into cysteine biosynthesis in *Acanthamoeba*. The data showed that recombinant CS actively catalyses the generation of cysteine (Fig. 3) and that the enzyme mRNA level is substantially downregulated when trophozoites were treated by abundant exogenous cysteine treated (Fig. 4 a, d). These results demonstrate that *Ac*CS participates in the synthesis of cysteine in *Acanthamoeba*. Sequence comparison (Additional file 1: Figure S1) revealed that *Ac*CS possesses all of the conserved substrate-binding sites but lacks the β8-β9 surface loop, a domain that is conserved in the CS associated with SAT [25–27]. This finding reveals that *Ac*CS does not form a regulatory complex with SAT in *Acanthamoeba* and is consistent with the lack of any evidence of a SAT homologue gene in the *A. castellanii* genome.

SPAT catalyses the first step of the non-phosphorylated serine metabolic pathway by transforming pyruvate to HP using serine as an amine donor [28]. GDH then catalyses HP reduction to glycerate, and glycerate kinase uses ATP as a phosphate donor to phosphorylate glycerate and produce 2-phosphoglycerate, which then enters the glycolytic pathway [29]. In the present study, we demonstrated that *A. castellanii* possesses SPAT and GDH in the non-phosphorylated serine metabolic pathway. The purified *Ac*SPAT had a specific activity of 13.17 ± 0.19 mmol/min per mg of protein for serine and showed a low *K*m when pyruvate was used as the substrate (Table 2). Such results indicate that *Ac*SPAT actively degrades L-seine to HP in *A. castellanii*. Moreover, the apparent *K*m of D-glycerate and NADP^+^ was higher than that of HP and NADPH, indicating the strong partiality of the GDH of this amoeba toward HP and NADPH as substrates compared with D-glycerate and NADP^+^. These data reveal that GDH may prefer to degrade, rather than generate, serine, consistent with the role of *Eh*GDH in *E. histolytica* [13]. *Ac*GDH and *Ac*SPAT colocalise in the inner part of the cell membrane and in the membranes surrounding the nucleus (Fig. 2), thus suggesting that they have similar physiological functions and that they can enter the nucleus of *Acanthamoeba* to participate in metabolic reactions. A parallel phenomenon, in which plant-like glycolytic enzymes can enter the nucleus, has been observed in *Toxoplasma gondii* [30].

The presence of the cysteine synthetic and non-phosphorylated serine metabolic pathways indicates that these protozoa have a high requirement for cysteine, perhaps for growth, attachment and pathogenicity, similar to its functions in *E. histolytica* [31] and *T. cruzi* [14]. In contrast to *E. histolytica* and *T. cruzi*, protozoans such as *Giardia intestinalis*, *Plasmodium falciparum* and *Cryptosporidium parvum* [6], lack both SAT and CS, perhaps because they do not have such a high requirement for cysteine or can absorb sufficient amounts of the amino acid from their hosts. Since different physiological functions exist in the cysteine metabolism pathway of protozoans, this metabolic pathway has been considered to be a potential drug target in some infectious diseases. For instance, in *Trichomonas vaginalis* and *E. histolytica*, CS is essential for cysteine synthesis from sulphide, and this parasitic enzyme could be an exploitable drug target because humans lack CS and the sulphur assimilation/cysteine biosynthetic pathway is absent from mammalian hosts [21, 31]. In *T. brucei*, an inhibitor of S-adenosylmethionine decarboxylase, was found to be highly effective against infections in mice [6]; methionine adenosyltransferase is also used therapeutically in *T. brucei rhodesiense* infections [32]. Moreover, *Plasmodium* S-adenosylhomocysteine hydrolase provides a practical target for antimalarial drugs [33]. However, in-depth investigations should be performed to confirm whether the cysteine synthetic or non-phosphorylated serine metabolic pathways could also be novel targets for the development of new drugs against acanthamoebiasis.

Unlike parasitic protozoa, free-living amoebae must immediately adapt to changing environmental conditions, such as starvation, increased osmolarity, extreme pH or temperature and oxygen depletion [2, 34]. Physiological features may be ascribed to the complex energy metabolism of trophozoites and cysts. *Acanthamoeba* trophozoites feed as aerobic protozoa in the presence of sufficient oxygen; however, under anoxic conditions, many free-living protists switch between aerobic and anaerobic metabolism [35]. *A. castellanii* possesses a complex energetic and respiratory system in which glycolysis [36] associated with aerobic mitochondria [37] and a hydrogenosomal-type anaerobic ATP generation pathway [38] function together to maintain its energy requirements. Moreover, *A. castellanii* prefers the anaerobic pathway for energy production [39], thus indicating that glycolysis may be an important energy metabolic pathway in *Acanthamoeba*. However, glycolysis does not always function throughout the life-cycle of *Acanthamoeba*. Fructose-bisphosphate aldolase and enolase are key enzymes in glycolysis that are repressed during *Acanthamoeba* encystation [40], which suggests that glycolytic enzymes are expressed stage-specifically. The same phenomenon has been observed in *Toxoplasma*, where enolase 1 is expressed in bradyzoites and enolase 2 is expressed in tachyzoites [41]. In *E. histolytica*, glucose-6-phosphate and fructose-6-phosphate are substantially depleted during encystation [42]. In our previous study, *Ac*PGDH and *Ac*PSAT were also expressed stage-specifically [16]. These enzymes were downregulated after treatment with L-serine or cysteine (Additional file 2: Figure S2), consistent with the *Ac*CS, *Ac*GDH and *Ac*SPAT expression observed in this study. Serine, in conjunction with pyruvate, is located at the glycolysis gateway [13]. Hence, we hypothesise that *Ac*GDH and *Ac*SPAT or *Ac*CS are involved in glycolysis regulation in *A. castellanii*.

*Acanthamoeba castellanii* hydrolyses glucose into pyruvate *via* glycolysis [34]. Glucose is a major precursor for cellulose synthesis [36], and many studies have demonstrated that cellulose synthesis is essential for cyst formation [43]. Thus, determining the role of glycolysis in cyst formation in *A. castellanii* is interesting [40, 44]. Analysis based on gene knockdown technology may provide an enhanced understanding of the roles of key enzymes, namely *Ac*GDH, *Ac*SPAT or *Ac*CS, during cyst formation. This knowledge may also provide an opportunity to explore drug targets, particularly the cyst formation of *A. castellanii*.

The pathogenicity of *Acanthamoeba* is related to its extracellular protease activities, and serine and cysteine proteases are the predominant extracellular proteases. Several serine and cysteine proteases have been identified in collagen degradation and corneal stroma invasion [2, 45]. Cysteine and serine are the catalytic residues at the active sites of these enzymes, and the influence of the activities of these enzymes on serine or cysteine biosynthesis has yet to be clarified. These observations, along with the fact that mammals do not perform sulphur assimilation/cysteine biosynthesis, indicate that the cysteine synthetic or nonphosphorylated serine metabolic pathway could be an attractive drug target for acanthamoebiasis treatment.

## Conclusions

The gene sequences of *Ac*CS, *Ac*GDH and *Ac*SPAT were elucidated in this study. *Ac*CS was involved in cysteine biosynthesis, whereas *Ac*GDH and *Ac*SPAT were involved in the non-phosphorylated serine degradation pathway and, therefore, in the glycolytic pathway of *A. castellanii*. This study is the first to provide molecular insights into the cysteine biosynthesis and non-phosphorylated serine metabolic pathways in free-living amoeba. Our results indicate the existence of the sulphur assimilation/cysteine biosynthetic pathway in the cysteine metabolism of *A. castellanii* and that *Ac*GDH and *Ac*SPAT may be involved in the energy metabolism, cyst formation and pathogenesis of *Acanthamoeba*. These findings could be further confirmed by studies using gene knockdown approaches or enzyme inhibitors. In summary, our data collectively suggest that *Ac*CS can be a target for the rational design of anti-*Acanthamoeba* drugs.

## Additional files

Additional file 1: **Figure S1.** Multiple alignments of amino acid sequences of CS from *A. castellanii* and other representative organisms. Sequences were aligned using AlignX (Vector NTI 11.5.3, Invitrogen). Sequences are as follows: Ac, *A. castellanii Ac*CS (this study); Bm, (*Balamuthia mandrillaris*, LEOU01001036); Dd, (*Dictyostelium discoideum*, XP629379); At (*Arabidopsis thaliana*, P47998); Eh (*E. histolytica*, BAA21916); Ei, (*Entamoeba invadens*, BAN42435); Lm (*Leishmania major*, CAJ09322); St (*Salmonella typhimurium*, AGQ86853); Tv (*Trichomonas vaginalis*, XP001325874). The letter X represents the uncertain sequences of *Dictyostelium discoideum* CS. Black shading indicates conserved amino acids. Arrowhead indicates active site lysine. Dots indicate binding sites for sulphur incorporation into cysteine. Arrows indicate key OAS-binding sites. Underlined domains indicate β8-β9 residues of CS that interact with SAT in *A. thaliana* and *S. typhimurium*. (TIF 6729 kb)

Additional file 2: **Figure S2.** Effect of cysteine on *PGDH* and *PSAT* gene expression in *A. castellanii* trophozoites. **a**, **b** Gene expression levels after treatment with different concentrations of cysteine (10, 20 and 40 mM) for 12 h. **c**, **d** Gene expression levels after treatment with cysteine for 24 h as a control, trophozoites were cultured in PYG medium without treatment. Vertical bars indicate SD. *P < 0.05, **P < 0.01 and ***P < 0.001 by one-way ANOVA. (TIF 1191 kb)

## Abbreviations

3-PHP: 3-phosphohydroxypyruvate
BMSA: *Babesia microti* merozoites
CS: Cysteine synthase
GDH: D-glycerate dehydrogenase
HP: Hydroxypyruvate
PGDH: Phosphoglycerate dehydrogenase
PSAT: Phosphoserine aminotransferase
SAT: Serine acetyltransferase
SPAT: Serine-pyruvate aminotransferase
