# Supplementary figures and images for: Molecular and biochemical characterization of key enzymes in the cysteine and serine metabolic pathways of Acanthamoeba castellanii
Source: Parasit Vectors. 2018 Nov 26;11:604. doi: 10.1186/s13071-018-3188-7 (PMC6257972; doi:10.1186/s13071-018-3188-7)

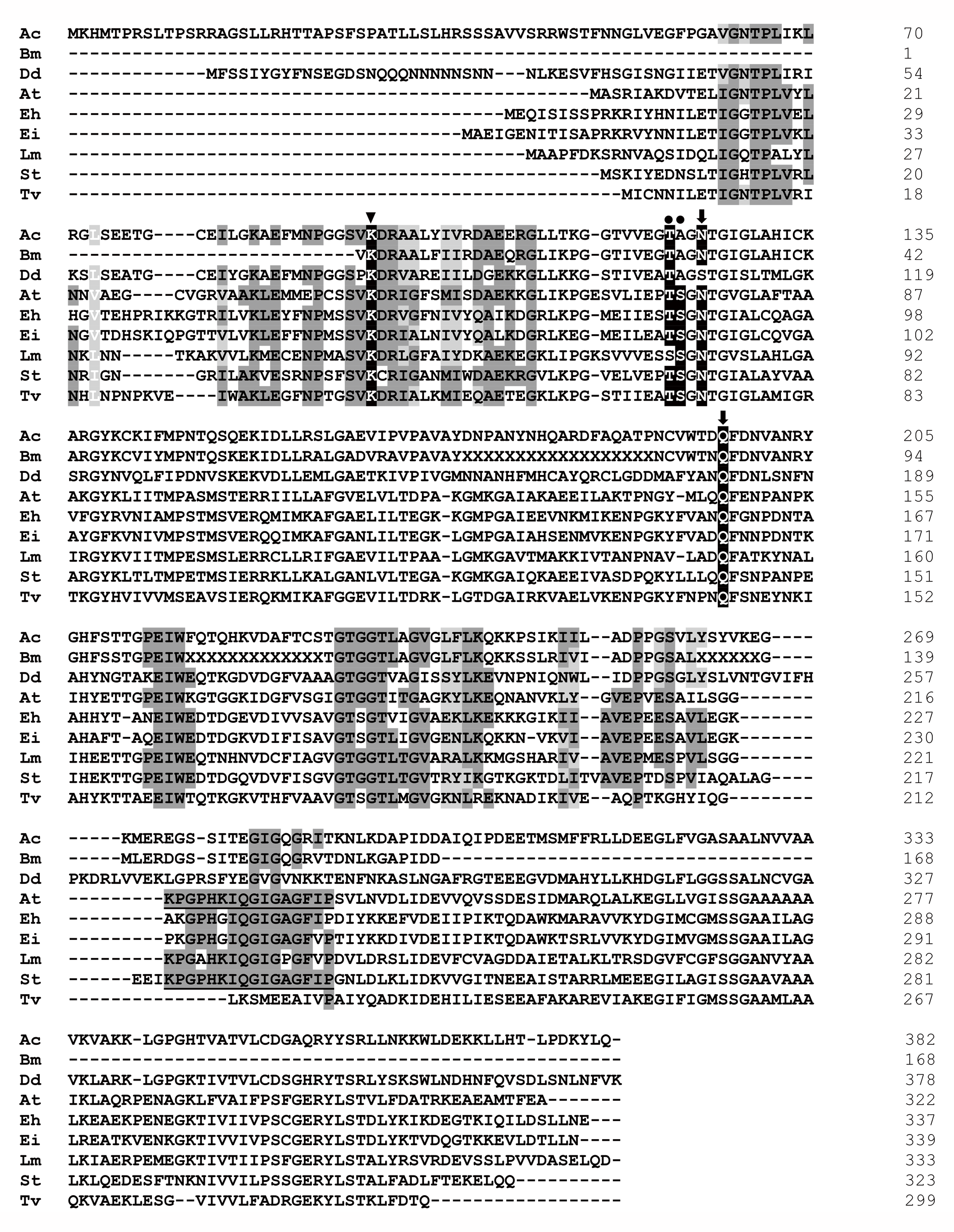

Supplement: Supplementary file 1 — Figure S1. Multiple alignments of amino acid sequences of CS from A. castellanii and other representative organisms. Sequences were aligned using AlignX (Vector NTI 11.5.3, Invitrogen). Sequences are as follows: Ac, A. castellanii AcCS (this study); Bm, (Balamuthia mandrillaris, LEOU01001036); Dd, (Dictyostelium discoideum, XP629379); At (Arabidopsis thaliana, P47998); Eh (E. histolytica, BAA21916); Ei, (Entamoeba invadens, BAN42435); Lm (Leishmania major, CAJ09322); St (Salmonella typhimurium, AGQ86853); Tv (Trichomonas vaginalis, XP001325874). The letter X represents the uncertain sequences of Dictyostelium discoideum CS. Black shading indicates conserved amino acids. Arrowhead indicates active site lysine. Dots indicate binding sites for sulphur incorporation into cysteine. Arrows indicate key OAS-binding sites. Underlined domains indicate β8-β9 residues of CS that interact with SAT in A. thaliana and S. typhimurium. (TIF 6729 kb) [file 13071_2018_3188_MOESM1_ESM.tif]

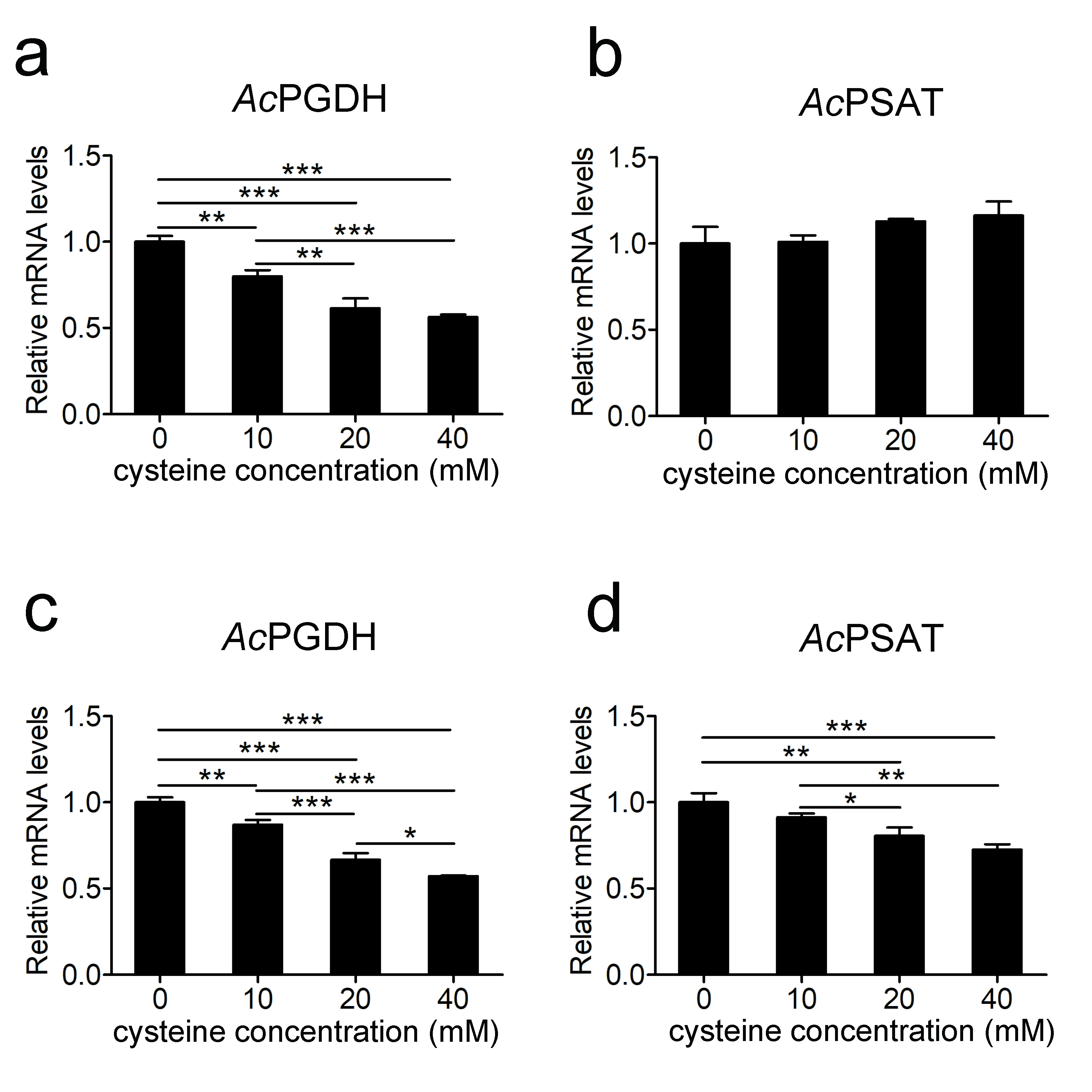

Supplement: Supplementary file 2 — Figure S2. Effect of cysteine on PGDH and PSAT gene expression in A. castellanii trophozoites. a, b Gene expression levels after treatment with different concentrations of cysteine (10, 20 and 40 mM) for 12 h. c, d Gene expression levels after treatment with cysteine for 24 h as a control, trophozoites were cultured in PYG medium without treatment. Vertical bars indicate SD. *P < 0.05, **P < 0.01 and ***P < 0.001 by one-way ANOVA. (TIF 1191 kb) [file 13071_2018_3188_MOESM2_ESM.tif]
